# Enhancing the Return to Work of Cancer Survivors: Development and Feasibility of the Nurse-Led eHealth Intervention Cancer@Work

**DOI:** 10.2196/resprot.5565

**Published:** 2016-06-10

**Authors:** Sietske J Tamminga, Sanne van Hezel, Angela GEM de Boer, Monique HW Frings-Dresen

**Affiliations:** ^1^ Coronel Institute of Occupational Health Academic Medical Center Amsterdam Netherlands

**Keywords:** cancer, return to work, eHealth, survivorship, feasibility studies, self-management

## Abstract

**Background:**

It is important to enhance the return to work of cancer survivors with an appropriate intervention, as cancer survivors experience problems upon their return to work but consider it an essential part of their recovery.

**Objective:**

The objective of our study was to develop an eHealth intervention to enhance the return to work of cancer survivors and to test the feasibility of the eHealth intervention with end users.

**Methods:**

To develop the intervention we 1) searched the literature, 2) interviewed 7 eHealth experts, 3) interviewed 7 cancer survivors, 2 employers, and 7 occupational physicians, and 4) consulted experts. To test feasibility, we enrolled 39 cancer survivors, 9 supervisors, 7 occupational physicians, 9 general physicians and 2 social workers and gave them access to the eHealth intervention. We also interviewed participants, asked them to fill in a questionnaire, or both, to test which functionalities of the eHealth intervention were appropriate and which aspects needed improvement.

**Results:**

Cancer survivors particularly want information and support regarding the possibility of returning to work, and on financial and legal aspects of their situation. Furthermore, the use of blended care and the personalization of the eHealth intervention were preferred features for increasing compliance. The first version of the eHealth intervention consisted of access to a personal and secure website containing various functionalities for cancer survivors blended with support from their specialized nurse, and a public website for employers, occupational physicians, and general physicians. The eHealth intervention appeared feasible. We adapted it slightly by adding more information on different cancer types and their possible effects on return to work.

**Conclusions:**

A multistakeholder and mixed-method design appeared useful in the development of the eHealth intervention. It was challenging to meet all end user requirements due to legal and privacy constraints. The eHealth intervention appeared feasible, although implementation in daily practice needs to be subject of further research.

**ClinicalTrial:**

Dutch Trial Register number (NTR): 5190; http://www.trialregister.nl/trialreg/admin/rctview.asp?TC=5190 (Archived by WebCite at http://www.webcitation.org/6hm4WQJqC)

## Introduction

Due to the improved survival rates for cancer in recent decades [1], remaining in or returning to work has become a relevant topic to address for people with a job when being treated for cancer. The number of people who have a job when diagnosed with cancer is expected to increase considerably in the coming years. This is mainly due to an increase in the retirement age, with the incidence of cancer within the working age group predicted to increase by 45% with the inclusion of people aged 65–70 years [1].

Research done over the past 10 years has indicated that cancer survivors are more likely than cancer-free controls to be unemployed [2,3] and they also experience problems upon their return to work [4]. These adverse work outcomes need to be improved through an appropriate intervention, as returning to work is considered a key survivorship issue by cancer survivors [5]. It also contributes to their quality of life and reduces financial problems [6]. A few interventions with the potential to enhance the return to work of cancer survivors have been studied in randomized controlled trials, with some interventions showing positive results on sustainable return to work, while others did not [7]. Of these interventions, multidisciplinary interventions showed the most promising results [7].

An overview of the outcomes of these above-mentioned intervention studies, as well as studies of factors predicting the return to work of cancer survivors, led to the following main insights. First, it is both feasible and appreciated by cancer survivors to address the issue of return to work in an early stage of psycho-oncological cancer care [8,9], although the amount of time available to spend on this topic is limited [9]. Second, self-assessed work ability is an important prognostic factor, irrespective of clinical characteristics [10]. Third, cancer survivors differ considerably in the time needed before their return to work [11] and the amount and timing of support required, suggesting that support needs to be tailored to the individual [12]. Fourth, employers are important stakeholders who can facilitate or hamper the return to work [13], while at the same time it appears difficult to establish cooperation between primary care, occupational care, and the workplace [9]. These main findings led to 2 hypotheses: 1) that an intervention aimed at enhancing the return to work of cancer survivors could be based on the theory of self-management, which can address misconceptions about self-assessed work ability through the technique of cognitive restructuring; and 2) that cooperation between specialist cancer care, the general practitioner, and occupational health care might be improved with integrative care management [14,15].

Based on the assumption that information and support need to be tailored to the individual, a stepped-care eHealth intervention may be a suitable solution. Other advantages of eHealth interventions include that they are easy to access and to tailor to the individual, both of which are important, as information on the Internet can be overwhelming [16]. Furthermore, eHealth interventions can be delivered interactively and also be integrated with traditional health care visits, which is much needed, given the limited availability of time in the health care system. Such eHealth approaches have also demonstrated that they are suitable for delivering self-management interventions for cancer survivors [17] and self-management interventions to enhance the return to work [18]. Finally, eHealth has also been used to facilitate involvement of the employer [14]. Nevertheless, a few drawbacks of eHealth interventions have been reported, such as difficulties with patients’ noncompliance, high dropout rates, and security and privacy issues [19,20].

Based on all of the above-mentioned findings, we decided to develop an intervention that would be part of psycho-oncological care and be based on the theory of self-management and integrative care management. We decided to deliver the intervention as stepped care through eHealth. In this paper, we report on this process, specifically addressing the above-mentioned challenges to eHealth interventions, including noncompliance.

The objectives of this study were to develop an eHealth intervention to enhance the return to work of cancer survivors and to test the feasibility of the eHealth intervention with end users.

## Methods

**Figure 1:**
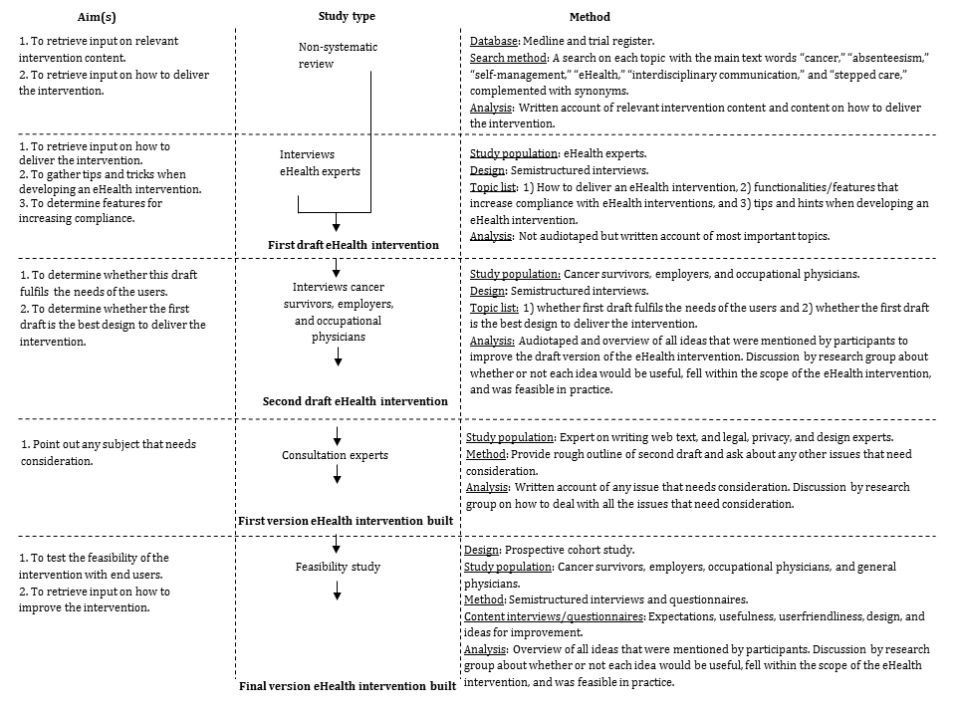
Flowchart of the development of Cancer@Work and the feasibility study.

We sought ethical approval to interview cancer survivors, employers, and occupational physicians, as well as approval for the feasibility study, from the Medical Ethics Committee of the Academic Medical Center, Amsterdam, the Netherlands, which judged that ethical approval was not required for either of the 2 studies (METC number W13_028 and W13_122). Participants (with the exception of experts) gave either their oral or written informed consent before the interview took place, or their written informed consent before participating in the feasibility study. We executed the following steps for objective 1 (Figure 1).

### Objective 1: Development of the Intervention Cancer@Work

We conducted a stepwise development of the intervention (Figure 1). We developed a first draft based on 1) a nonsystematic literature review and 2) interviews with experts in an iterative manner. Thereafter, we undertook 3) semistructured interviews with end users (cancer survivors, employers, occupational physicians) to test whether this first draft fit the needs of the users and whether it was the best design to deliver the intervention. Subsequently, we adapted the first draft on the basis of the results of these interviews. Using this second draft, we built a first version of the website after 4) consultation with experts, including a legal expert.

#### Nonsystematic Literature Review

##### Methods

Our literature search was focused on self-management, integrative care management, stepped care, and eHealth in a nonsystematic manner. The aim of this search was to retrieve input on relevant intervention content and input on how to deliver the intervention. We restricted our search to MEDLINE, searching the trial register for relevant interventions. The search was carried out between March and May 2012. We completed a search for each topic using the main text words: “cancer,” “absenteeism,” “self-management,” “eHealth,” “interdisciplinary communication,” and “stepped care,” complemented with synonyms. We also searched references from relevant studies. The results of our search were analyzed on the basis of a written account of the relevant intervention content and relevant information on how to deliver the intervention.

##### Results

Several studies described self-management interventions that aimed to improve individuals’ skills and confidence, helping them deal with their disease more effectively in daily life [21]. Research on self-management has shown that it has positive effects in cancer survivors dealing with survivorship issues [22], as well as in chronically ill patients dealing with work-related issues [23]. The key elements of self-management interventions are information and assignments. Self-management interventions can be based on problem-solving techniques or cognitive behavioral techniques, and have proven to be equally effective in treating patients with depression [24]. When addressing misconceptions about cancer and work, a cognitive behavioral component seems most appropriate, since the core focus of this technique is on cognitive reframing. In addition, to resolve a patient’s work-related problems, a problem-solving component seems most appropriate, since this technique attempts to resolve patients’ problems by teaching them structured problem-solving skills and how to generate achievable and simple action plans [26]. Moreover, one self-management intervention based on a problem-solving technique aimed at increasing the work functioning of employees with rheumatoid arthritis demonstrated its feasibility and was appreciated by both patients and care providers [27]. We therefore decided to include both components, offering problem-solving and cognitive behavioral techniques.

A few studies have tried to enhance collaboration between primary and occupational health care, but the level of effectiveness is still inconclusive. Some studies have shown positive results with respect to feasibility [28] and effectiveness [15], but others did not demonstrate any positive results [9,29]. One strategy that did prove feasible is integrative care management [15]. The goal of integrative care management is to establish the much-needed collaboration between all stakeholders (eg, the sick-listed employee, the occupational physician, the employer, the general physician, and the treating physician) to achieve a successful return to work of the sick-listed employee. A coordinator is ideally situated to establish this collaboration. However, collaboration can also be established using a secure website, where stakeholders can work together toward a common goal and share the necessary information to achieve this. In the eHealth intervention developed by Vonk Noordegraaf et al [14], almost half of the participants invited their supervisor to be involved in the eHealth intervention and that approximately 60% of the supervisors took this opportunity [30].

In a stepped-care intervention, all patients start with a low-intensity intervention, with the intensity increasing only if an intervention goal is not achieved at a certain point in time (eg, [31]). The advantages of a stepped-care intervention include the ability to deliver the right intensity of an intervention to each patient, lower intervention costs, and less time wasted by patients who participate [32]. The challenge for stepped-care intervention, however, is determining the cutoff value that indicates whether the intervention goal has been met and when a judgment can be made about whether the intervention goal has been met. When using a self-management intervention based on problem-solving techniques, it has been proposed that patients should be carefully selected to reduce noncompliance and dropout [27]. For this reason, a self-management intervention based on problem-solving techniques seems relevant only in cases where a patient experiences problems with his or her return to work, and it should therefore be offered only as a second step in an intervention.

Several eHealth intervention features are related to enhanced patient compliance: peer and counsellor support, email and phone contact, updates, record keeping, and individualized feedback [33,34]. Furthermore, eHealth interventions that are combined with face-to-face contact with a health care professional also showed enhanced patient compliance [35].

#### Interviews With Experts on eHealth Interventions

##### Methods

We approached approximately 10 experts based on the fact that they had recently developed an eHealth intervention for cancer survivors or an eHealth intervention aimed at increasing return to work of sick-listed employees. The first author (ST) undertook semistructured interviews based on a topic list. This list consisted of questions on 1) how to deliver an eHealth intervention, 2) features required to increase compliance with eHealth interventions, and 3) tips and tricks for developing an eHealth intervention. The interviews were held in August 2012. They were not audiotaped but the first author (ST) provided a written summary of the most important issues mentioned during the interview.

##### Results

In total, 7 eHealth experts were interviewed with the aim of 1) gaining input on how to deliver the intervention, 2) gathering tips and tricks for developing an eHealth intervention, and 3) determining features that increase compliance. These eHealth experts reported that the following elements were required to deliver an eHealth intervention: choose a modular or a structured form; and use a combination of text, film, and pictures. The following additional features that increase compliance with eHealth interventions were also suggested: sending reminders, and using blended care with a health care professional. Finally, the experts also noted that what was most important when developing an eHealth intervention was 1) to consider, before starting, what type of “track and trace data” would be required to study use and compliance, and 2) to consult an information technology expert at the beginning of the development of the intervention because they can give advice at an early phase about the functionalities that are possible given financial and practical constraints. As return-to-work trajectories vary across patients in terms of length [36] and needs, we decided to deliver the eHealth intervention in a modular form. As we considered the remainder of the above-mentioned suggestions to be valuable for our eHealth intervention, we decided to use them all. Based on the literature search and interviews with these experts, we created a first draft of the intervention (Multimedia Appendix 1,Multimedia Appendix 2,Multimedia Appendix 3).

#### First Draft

This first draft of the eHealth intervention consisted of access to a personal, tailored, and secure website containing various functionalities for cancer survivors, blended with support from their nurse, occupational physician, and employer (Multimedia Appendix 1,Multimedia Appendix 2,Multimedia Appendix 3). We presented this first draft as an online working environment. Blended care delivered by specialized nurse encompassed 1) answering questions, 2) monitoring and supervising use of the eHealth intervention, 3) providing personal feedback on assignments on the eHealth intervention, and 4) encouraging patients to comply with the intervention. Functionalities included providing insight into laws and regulations, a library, and a self-test to gain insight into opportunities to return to work (based on the problem-solving technique component), and redressing negative ideas concerning the possibilities to work after cancer, including the idea that one can return to work only after complete recovery (based on cognitive behavioral technique components). The occupational physician and employer had access to a specific part of the secure website that contained certain information, allowed them to access the cancer survivor’s return-to-work plan, and allowed them to add their suggestions to facilitate their employee’s return to work.

#### Interviews With Cancer Survivors, Employers, and Occupational Physicians

##### Methods

Cancer survivors were eligible to participate if they had been working at the time of diagnosis, were fluent in Dutch, and were aged between 18 and 65 years. Occupational physicians and employers were eligible to participate if they had recently encountered an employee with cancer and were fluent in Dutch. We intended to interview 10 cancer survivors, 5 employers, and 5 occupational physicians. The first author (ST) undertook semistructured interviews after the interviewees had been given a rough outline of the first draft of the intervention. They were asked whether this first draft accorded with their needs and whether they thought the design was the most appropriate to deliver the intervention. The interviews were held in March 2013; they were audiotaped but not transcribed verbatim. We analyzed the interviews using a direct content analysis [37] in order to obtain an overview of all ideas mentioned by the participants that could improve the draft version of the intervention. Subsequently, 3 of the authors (ST, AdB, MF) discussed whether each idea would be useful, whether it fell within the scope of the intervention, and whether it was feasible in practice (eg, financial constraints).

##### Results

In total, we interviewed 7 cancer survivors, 2 employers, and 7 occupational physicians with the aim of determining whether this draft fulfilled the needs of the users and whether it was the best design for delivering the intervention. We added the following functionalities to the first draft for cancer survivors: assignments providing insight into possible financial consequences, a documentary called *Irrevocable*, and assignments providing insight into the individual importance of work (Multimedia Appendix 1). We added the assignments providing insight into possible financial consequences and the individual importance of work because cancer survivors mentioned that they would have benefited from such insight and that this information is not readily available. In addition, to meet the needs of cancer survivors for peer support, and given our financial constraints, we added the documentary, which follows the return-to-work trajectories of 3 cancer survivors. For employers, we added a functionality that addressed the need to involve colleagues. Finally, in relation to occupational physicians, we added advice for cancer survivors on how to remain in contact with their workplace and references to relevant guidelines. We altered or removed some functionalities of the first draft of the eHealth intervention (eg, the occupational physician’s and employer’s access to a specific part of the secure website containing certain information, and the possibility to see the cancer survivor’s return-to-work plan and add their suggestions to facilitate their return to work). In addition, we excluded some suggested functionalities from the eHealth intervention due to legal, privacy, financial, or practical constraints (eg, the ability to obtain peer support from former cancer survivors) or because we did not consider them to be of added value (eg, teaching the user how to deal with the expectations of others) or within the scope of the intervention (eg, information on benefits from municipalities and on the long-term disability pension) (Multimedia Appendix 1). Based on these interviews, we developed a second draft (Multimedia Appendix 1,Multimedia Appendix 2,Multimedia Appendix 3).

#### Second Draft

The second draft consisted of access to a personal and secure website containing various functionalities for cancer survivors blended with support from their nurse, and a public website for occupational physicians, general physicians, and employers (Multimedia Appendix 1,Multimedia Appendix 2,Multimedia Appendix 3). Blended care delivered by specialized nurse encompassed 1) answering questions, 2) monitoring and supervising use of the eHealth intervention, 3) providing personal feedback on assignments on the eHealth intervention, and 4) encouraging patients to comply with the intervention. Functionalities for patients included support to draw up a strategy to manage specific personal problems that might inhibit their return to work, assignments providing insight into possible financial consequences of being on sick leave, support to draw up a return-to-work plan, and how to redress negative ideas concerning the possibilities of working after cancer, including the idea that one can return to work only after complete recovery (based on cognitive behavioral technique components).

#### Consultation With Experts on Writing Web Text and Website Design, Privacy, and Legal Aspects of Building an eHealth Intervention

##### Methods

We consulted an expert on writing text for websites and an expert on design, privacy, and legal aspects of building an eHealth intervention. They were given the rough outline of the second draft of the intervention and were asked to point out any issues that required further consideration.

##### Results

Consultation with an expert on writing Web text taught us that it is important to 1) draft a “tone of voice document,” which reflects the key values of the intervention, and on the basis of which the text is written, 2) to write clear and concise sentences without ambiguity, and 3) to develop the website in such a way that it is personal, for example, by using quotes from former cancer survivors that include their name, age, and occupation. The key values for this intervention were reliability, activating, personal, confrontational, serious, and directed to work and income. The design expert showed us that it is essential to match the design of the website with the tone of voice and Web text. Based on the tone of voice we wanted, the design expert created style sheets (Multimedia Appendix 4,Multimedia Appendix 5). After consultation with privacy and legal experts, it appeared that some functionalities would not be possible due to privacy legislation and that we needed to add a disclaimer pointing out the risk of using two functionalities and provide more secure alternatives (Multimedia Appendix 1,Multimedia Appendix 2,Multimedia Appendix 3).

#### First Version

Based on consultations with these experts, we built a first version of the eHealth intervention, consisting of access to a personal and secure website containing various functionalities for cancer survivors blended with support from their nurse, and a public website for occupational physicians, general physicians, and employers (Multimedia Appendix 1,Multimedia Appendix 2,Multimedia Appendix 3). Functionalities for patients included support in gaining insight into the individual importance of work, the ability to invite their employer, occupational physician, or general practitioner to use the public website (as a first step), and guidance in drawing up a strategy to manage specific personal problems that might inhibit their return to work (as a second step). Cancer survivors and specialized nurses were given a unique username and password to log in to the secure webpage, while verification would take place by text message.

### Objective 2: Feasibility of the Intervention Cancer@Work

After developing the first version of the intervention, we tested its feasibility by interviewing 1) cancer survivors, 2) employers, occupational physicians, and general physicians, and 3) social workers. All of the interviews discussed below were audiotaped but not transcribed verbatim. We intended to interview 40 cancer survivors, 10 employers, 10 occupational physicians, and 10 general physicians. We analyzed the interviews using a direct content analysis [37] in order to obtain an overview of all of the ideas that were mentioned by participants that might improve the first version of the eHealth intervention. Subsequently, we discussed whether each idea would be useful, whether it fell within the scope of the eHealth intervention, and whether it was feasible in practice. The cancer survivors also filled in 2 questionnaires.

#### Feasibility of the Intervention for Cancer Survivors

##### Methods

To test the feasibility of the intervention for cancer survivors, 1) we invited cancer survivors who had taken part in previous research by our department and who had given their consent to be approached in future research on cancer and work, and 3) the treating physician also invited cancer survivors who were treated between 2012 and 2014 in the department of gynecological oncology of an academic medical center. Cancer survivors were eligible to participate if they had been working at the time of diagnosis, were fluent in Dutch, and were aged between 18 and 65 years. The patients were sent an informative letter about the study. If interested, they could return a consent form agreeing to telephone contact, whereupon 1 of the researchers contacted the patient by phone. During this conversation they could ask questions and decide whether they wanted to participate.

We used the following criteria to assess feasibility: 1) appropriateness of each functionality, 2) which functionalities needed improvement, 3) usefulness of the eHealth intervention, 4) user friendliness of the eHealth intervention, and 5) whether the eHealth intervention met their expectations. We considered the eHealth intervention feasible when at least 50% of the patients responded positively to each criterion.

After patients gave their informed consent, they filled in a questionnaire on their expectations of the intervention (eg, “What are your expectations regarding the Internet program Cancer@Work?”) and their need for support regarding their return to work (eg, “Do you need support on questions or problems regarding your work?”). After completing the questionnaire, they were given access to the eHealth intervention for 6 weeks, after which they filled in another questionnaire about the usefulness and user friendliness of the intervention (eg, “Which functionalities from Cancer@Work did you find useful?”) and whether the intervention met their expectations (eg, “Did Cancer@Work meet your expectations?”; “Cancer@Work did (not) meet my expectations because...”). All of the participants who filled in both questionnaires were invited to participate in a telephone interview by 1 of the researchers (SvH) to potentially gain greater insight into the answers to the questionnaire and to ask whether they had any further suggestions to improve the eHealth intervention and its functionalities. These interviews were held in June 2015. Based on these interviews, we developed a final version of the eHealth intervention for cancer survivors.

##### Results

Of the 177 cancer survivors invited to participate, 39 (22.0%) filled in the first questionnaire, while 20 filled in the second questionnaire (each participant was contacted by phone to remind them to fill in the questionnaire). We used the data of the 20 participants who filled in both questionnaires for our analysis. Table 1 details the participants’ characteristics. The main reason that 19 participants did not fill in the second questionnaire was that they did not feel the need to use the intervention because it was, on average, 3 years since they had received their cancer diagnosis and they had already returned to work.

**Table 1:** Characteristics of cancer survivors who participated in the feasibility study (n=20).

Variables				n (%) or mean (SD)	
**Sociodemographic variables**					
	Female, n (%)			18 (90)	
	Age in years, mean (SD)			49 (10)	
	**Marital status, n (%)**				
		Married/living together		11 (55)	
		Single		5 (25)	
		Divorced		4 (4)	
	**Educational level, n (%)**				
		Lower vocational education		1 (5)	
		Secondary vocational education		8 (40)	
		Intermediate vocational education		1 (5)	
		Higher professional education		6 (30)	
		University		4 (20)	
	**Cancer diagnosis, n (%)** ^a^				
		Breast cancer		8 (40)	
		Gynecological cancer		7 (35)	
		Lymphoma		1 (5)	
		Bowel cancer		2 (10)	
		Skin cancer		1 (5)	
		Hematological cancer		1 (5)	
	**Cancer treatment, n (%)** ^a^				
		Surgery		17 (85)	
		Chemotherapy		14 (70)	
		Radiotherapy		9 (45)	
		Hormonal therapy		7 (35)	
		No treatment		2 (10)	
**Work-related variables**					
	**Current work status, n (%)**				
		Fully returned to work		16 (80)	
		Partially returned to work		1 (5)	
		Fully sick listed		1 (5)	
		Never sick listed		1 (5)	
		Data missing		1 (5)	
	**Type of occupation, n (%)**				
		Physical heavy work		2 (10)	
	**Type of employment contract, n (%)**				
		Permanent employment contract		15 (75)	

^a^Numbers do not add up because of possibility of giving multiple answers.

**Table 2:** Results of questionnaire for cancer survivors who participated in the feasibility study (n=20).

Intervention-related questions		n (%)
**Need for information on cancer and work**		
	A bit to very much	9 (45)
**Need for support on cancer and work**		
	A bit to very much	7 (35)
**Are you experiencing problems with returning to work?**		
	Yes	6 (30)
**Reason for not using eHealth intervention Cancer@Work**		
	No need	4 (20)
	Not up to it yet	1 (5)
	No time	1 (5)
	Forgot to log in	1 (5)
	Other	3 (15)
**Reason for using eHealth intervention Cancer@Work**		
	Useful program	1 (5)
	My specialized nurse encouraged me to use Cancer@Work	0 (0)
	My supervisor/occupational physician encouraged me to use Cancer@Work	0 (0)
	Other	10 (50)
**Did Cancer@Work meet your expectations?**		
	Yes	1 (5)
	Yes, a little	3 (15)
	No, not at all	1 (5)
	Do not know/did not have any expectations	15 (75)
**Reason why Cancer@Work met my expectations**		
	Receive information on cancer and work	2 (10)
	Receive support for work-related problems	0 (0)
	Get insight into my work-related problems	0 (0)
	Get solutions to work-related problems	0 (0)
	Get help from others	0 (0)
	Other	2 (10)
**Reason why Cancer@Work did not meet my expectations**		
	Did not receive information on cancer and work	0 (0)
	Did not receive support for work-related problems	0 (0)
	Did not get insight into my work-related problems	0 (0)
	Did not get solutions for work-related problems	0 (0)
	Did not get help from others	0 (0)
	Other	1 (5)
**Did Cancer@Work fit into daily use?**		
	Yes or somewhat	2 (10)
**For whom is Cancer@Work appropriate?** ^a^		
	For me	2 (10)
	For all cancer survivors with a job	9 (45)
	For all cancer survivors with cancer-related problems	13 (65)
	For cancer survivors who lost their job	8 (40)
	Do not know/no opinion	2 (10)
	Other	5 (25)
**Is Cancer@Work useful?**		
	Very useful	5 (25)
	Useful	10 (50)
	Not useful	5 (25)
	Do not know/no opinion	0 (0)
**Is Cancer@Work user friendly?**		
	Yes	12 (60)
	No	1 (5)
	Do not know/no opinion	7 (35)
**Is it useful to be able to ask for support with the use of Cancer@Work?**		
	Yes	5 (25)
	No	3 (15)
	Do not know/no opinion	12 (60)

^a^Numbers do not add up because of possibility of giving multiple answers.

In total, 11 participants (55%) used the eHealth intervention. The other participants did not use the intervention either because they forgot to log in or they did not have any problems at work and thus did not feel the need to use the intervention. Table 2 details the results of the questionnaires. One of the participants’ expectations regarding Cancer@Work were not met because she had already fully returned to work and did not have any work-related problems. Furthermore, 1 participant did not find the eHealth intervention user friendly because there was too much theoretical information and not enough opportunities to interact with other cancer survivors.

All of the 20 participants were invited for a telephone interview and 11 participated. The other 9 participants did not participate because of time constraints, practical issues, or lack of interest. During the interviews, the participants were positive about the intervention and its functionalities. They proposed several ideas to improve the intervention, such as making it easier to log in by using less-complicated passwords or not using text message authentication, or to make the intervention accessible for vision-impaired people by making it possible to enlarge the size of the text or to have the text read aloud. For an overview of all of the ideas that were proposed to improve the intervention, see Multimedia Appendix 1,Multimedia Appendix 2, and Multimedia Appendix 3. We did not use any of these ideas to improve the intervention in the final version, either because they did not meet the security or privacy regulations or because of practical constraints. Therefore, we made no changes and the first version became the final version of the intervention for cancer survivors.

#### Feasibility of the Intervention for Employers, Occupational Physicians, and General Physicians

##### Methods

Employers were eligible to participate if they had recently encountered an employee with cancer, while occupational physicians and general physicians did not need to have recent experience with a cancer patient or the associated work-related problems. All of the participants had to be fluent in Dutch. After giving informed consent, participants visited the eHealth intervention website designed for their respective profession and then participated in a semistructured interview to gather information on participants’ experience with the intervention, its usefulness, and its user friendliness. The interviews were conducted in January and February 2015 by 1 of the authors (SvH) and they were audiotaped but not transcribed verbatim. Based on these interviews, we developed a final version of the intervention for employers, occupational physicians, and general practitioners.

##### Results

In total, 9 employers, 7 occupational physicians, and 6 general physicians participated in the interviews. For a full overview of the ideas that were mentioned, see Multimedia Appendix 1,Multimedia Appendix 2, and Multimedia Appendix 3. The main issue that arose from the interviews was that participants needed more detailed information, mainly on the different cancer diagnoses and treatments, and their possible effects on the employee’s ability to return to work. The occupational physicians were least enthusiastic about the intervention, since they felt that they already had a lot of knowledge on this topic. Thus, the occupational physicians in particular indicated that more detailed information was required to make the intervention useful. As a result, in the final version of the intervention, we added more detailed information on different cancer diagnoses and treatments and their possible effects on the employee’s ability to return to work. In addition, we added many institutions to the list of support agencies they could consult.

When asked about the user friendliness, design, and practical usefulness of the intervention, almost all of the participants indicated that the intervention was easy to use (n=21, 96%), that the design matched the content and purpose of the website (n=20, 91%), and that they would recommend this intervention, for example, to their friends or colleagues if they had to deal with an employee, client, or patient with cancer (n=19, 86%). Thus, we made no changes regarding these aspects in the final version of the intervention (see Multimedia Appendix 1,Multimedia Appendix 2,Multimedia Appendix 3).

#### Feasibility of Blended Support From the Cancer Survivor’s Specialized Nurse

##### Methods

We asked 2 social workers from a gynecology department of a Dutch hospital who had experience supporting cancer survivors in their return to work to visit the section of the eHealth intervention for specialized nurses and to participate in a semistructured interview with 1 of the authors (SvH). Based on this interview, we developed a final version of the section of the eHealth intervention for specialized nurses.

##### Results

A total of 2 social workers participated in 1 interview, on the basis of which we added information on financial and legal issues to the final version of the intervention in order to provide specialized nurses and social workers with access to the same information as that provided to the patients they are supporting (Multimedia Appendix 1,Multimedia Appendix 2,Multimedia Appendix 3). In this way, it would be easier for them to advise patients on financial and legal issues by referring to the intervention. Furthermore, the social workers indicated that the intervention was easy to use, and that they anticipated no problems in using the intervention in practice.

## Discussion

The objectives of this study were to develop an eHealth intervention to enhance the return to work of cancer survivors and to test the feasibility of the eHealth intervention with end users. The results of our development study show that cancer survivors particularly want to receive information and support on opportunities to return to work, and on the financial and legal aspects of their position. Furthermore, we found that the use of blended care and the personalization of the eHealth intervention are preferred features for increasing compliance. The results of the feasibility study showed that the eHealth intervention was feasible; however, we adapted it slightly by adding more information on different types of cancer and its treatment and on the possible effects of these on the return to work.

### Strengths and Limitations

The strengths of our study include the use of a multistakeholder and mixed-method design to develop our eHealth intervention. In this way, the content, design, and delivery of the eHealth intervention could best fulfill the needs of the end users and be the most suitable option to achieve the intervention goal, given Dutch law and our framework of psycho-oncological care. This also ensured that the risk of a type III error (ie, theory failure) was minimized [38].

We did not test the feasibility of the eHealth intervention in precisely the same conditions under which the intervention is intended to be used, as the feasibility study among cancer survivors was a prospective cohort study, with a short follow-up period of 6 weeks, that started on average 3 years after diagnosis, while the eHealth intervention is intended to be used from the initial cancer diagnosis until sustainable return to work. As a result, we are unable to draw firm conclusions about the usefulness of the content of the eHealth intervention, the features that enhance compliance, and the features that enhance integrative care management. We have partly overcome this drawback by consulting the literature, and interviewing experts and end users under our first objective. However, implementation of the eHealth intervention in daily practice should be the subject of further research. We were able to test the appropriateness of all the functionalities and the user friendliness, the usefulness, and the design of the eHealth intervention, for which we found positive results, so that the criteria for feasibility were met.

The employers, occupational physicians, and general practitioners who participated in the interviews and feasibility study were generally very interested in this subject for personal reasons, for example, as a cancer survivor. This was very useful for generating ideas about the content of the eHealth intervention, but it might have led to an overestimation of its future use by other members of their profession in daily practice. Implementation in daily practice should, for this reason, also be the subject of further research.

### Interpretation of Findings

Cancer survivors, employers, occupational physicians, general practitioners, and specialized nurses were generally positive about the eHealth intervention. Not surprisingly, occupational physicians saw the least added value of the eHealth intervention for their own profession, as they thought that they were already applying the content in their daily practice. However, in their experience, they believed that employers would particularly benefit from the content, as they rarely have to deal with an employee with cancer, and cancer remains a difficult topic to address in the workplace [13,39]. We also added specific content to the eHealth intervention for general practitioners, for two reasons. First, patients themselves wanted more return-to-work guidance from their general practitioner, in addition to guidance from their occupational physician or in the absence of an occupational physician [25]. Second, general practitioners play a more prominent role in psycho-oncological care after primary treatment, including dealing with the possible effects on social outcomes such as being able to return to work [40]. We hope that we have equipped general practitioners with sufficient information and referral options. Apart from these issues, it seems important that future research examine what general practitioners specifically require to support cancer survivors in their return to work.

Although 97% of Dutch society has Internet access [41], Internet illiteracy is associated with a lower educational level [41]. At the same time, research indicates that self-management interventions are more effective in patients with a lower educational level [23]. Moreover, cancer survivors with a lower educational level are less likely to return to work [42], indicating that the intervention might be especially needed among this group. For these reasons, we spent additional time and effort to ensure that the eHealth intervention was very easy to use so that people with limited Internet literacy would be able to use the eHealth intervention as well. As appeared from both our quantitative study and our qualitative study, where we found that participants had very few problems using the eHealth intervention, we might have succeeded in this goal.

Due to legal regulations, we were obliged to add a disclaimer to two functionalities explaining the risk of using each of them and suggesting a more secure alternative, for example, in relation to inviting their employer, occupational physician, or general practitioner to view personal information via an email. The use of email is generally not considered a secure way to exchange information if you wish to keep the information completely confidential. Although we fully agree that it is our responsibility to inform patients about any possible harm, such warnings might cause unnecessary concern and discourage them from using the eHealth intervention at all, which might lead to otherwise preventable dropout. We therefore recommend that researchers engage the services of a legal adviser from the start when developing an eHealth intervention.

The process evaluation of the study by Bouwsma et al [30], which investigated an interactive eHealth intervention on the return to work of gynecological patients with benign tumors, showed that only half of the participants invited their employer to visit an anonymous section of the website. Most participants reported that the reason for not using this tool was “finding it unnecessary because of fast recovery and good relationship with employer” [30], while most employers reported being satisfied or very satisfied with the information provided [30]. As the return-to-work trajectories of patients with malignant tumors are significantly longer than for patients with benign tumors (median sick-leave days 102 vs 44) [43,44], we expect that there will be more substantial need to use this functionality among our population. We therefore decided to include a comparable functionality without alteration.

### Implications for Further Research

One subject of further study should be the feasibility of the eHealth intervention in daily practice, and such a study should especially focus on the functionalities aimed at increasing compliance and integrative care, as we were unable to study them this feasibility study. In addition, the effectiveness of the eHealth intervention on a sustainable return to work should also be further studied, and the intermediate effect of the eHealth intervention on self-management skills and work-related self-efficacy should be analyzed (ie, a measure to study a change of ideas regarding possibilities to return to work). We are studying both of these in a multicenter randomized controlled trial.

### Conclusion

A multistakeholder and mixed-method design appeared useful in the development of the eHealth intervention. However, it is challenging to meet all end users’ requirements due to legal and privacy constraints. The eHealth intervention appeared feasible, although implementation in daily practice requires further research.

## Appendix

Multimedia Appendix 1 Functionalities of Cancer@Work at different stages of development.

Multimedia Appendix 2 Design of Cancer@Work at different stages of development.

Multimedia Appendix 3 Features to increase compliance with Cancer@Work at different stages of development.

Multimedia Appendix 4 Screenshot from the eHealth intervention - quiz.

Multimedia Appendix 5 Screenshot from the eHealth intervention - home page.
