# Supplementary material for: Enhancing the Return to Work of Cancer Survivors: Development and Feasibility of the Nurse-Led eHealth Intervention Cancer@Work
Source: JMIR Res Protoc. 2016 Jun 10;5(2):e118. doi: 10.2196/resprot.5565 (PMC4920959; doi:10.2196/resprot.5565)
Supplement: Multimedia Appendix 1 [file resprot_v5i2e118_app1.pdf]

Multimedia Appendix 1. Functionalities of Cancer@Work in different stages of development.

**Functionality**		**Draft 1** – Based on literature review and interviews with eHealth ts	**Draft 2 –** Adapted on the basis of interviews with cancer survivors, employers and occupational physicians	**First version of website build –** after consultation with experts on writing web text and experts on design, privacy and legal aspects	**Final website** adapted on the basis of results feasibility study
**Cancer patients** Access to personal and secure website	Library on cancer and work	√	√	√	√
	Frequently asked questions by cancer survivors about cancer and work	√	√	√	√
	Diary and logbook	√	√	√	√
	Gain an insight into possible financial consequences of sick leave	-	√	√	√
	Gain an insight into rights and obligations according to the Dutch social security system when sick-listed	√	√ But tailored to cancer patient's work situation[b]	√	√
	The documentary, *Irrevocable*, in which three cancer survivors are followed in their return-to-work trajectories	-	√	√	√
	Gain an insight into individual importance of work	-	√	√	√
	Draw up return-to-work plan	√	√	√	√
	Self-test to gain insight into opportunities to return to work and restructuring of cognition	√	√	√	√
	Quotes from former cancer patients	√	√	√ But personalised	√
	Invite employer, occupational physician and general practitioner to visit public website on cancer and work	-	√	√ But with disclaimer stating that stakeholder will know that patient is diagnosed with cancer	√
	Send message to email address of their employer, occupational physician, and general practitioner[a]	-	√	√ But with disclaimer stating that use of email is not secure and that it is safer to contact stakeholder face-to-face or by telephone	√
	Draw up strategy to manage specific personal problems that might inhibit their return to work – offered as second step of intervention	√	√	√	√
	Support from former cancer patients	-	Excluded due to financial constraints	-	-

	Invite employer and/or occupational physician to see their return-to-work plan and add suggestions to facilitate their return to work	√	Excluded due to privacy/legal constraints	-	-
	To inform occupational physician about cancer diagnosis and cancer treatment	-	√	Excluded due to legal constraints	-
	Support from reintegration specialist	-	Excluded due to financial constraints	-	-
	Information on benefits from municipalities and on long-term disability pension	-	Excluded as outside the scope of eHealth intervention	-	-
	Register a patient face-to-face, including an extensive work anamnesis	-	Excluded as not feasible in cancer care	-	-
	Deliver information dependent on cancer diagnosis and treatment	-	Excluded due to financial constraints	-	-
	Convalescence recommendation based on cancer diagnosis and treatment	-	Excluded because such recommendations depend on too many other variables	-	-
	Teach that communication is very important	-	Excluded because not expected to directly benefit return to work	-	-
	Teach how to deal with expectations of others	-	Excluded because not expected to directly benefit return to work	-	-
	Logging in without the use of SMS authentication	-	-	-	NA: because of privacy and security regulations
	Using less complicated passwords to sign in	-	-	-	NA: because of privacy and security regulations
**Employers** Public website	Discuss disclosure about cancer diagnosis with employee	-	√	√	√
	Involve colleagues in return-to-work process of employee with cancer and create understanding among colleagues about the situation of sick-listed employee	-	√	√	√ But extended
	Information on how to counsel employee with cancer, including dos and don'ts	√	√	√	√
	General information on cancer diagnosis and treatment	-	-	-	√
	Information on possible effects of cancer diagnosis/treatment on return to work	√	√	√	√ But extended
	Ask patient's specialised nurse	-	√	Excluded due to	-

	questions			privacy legislation	
	Information on financial consequences for employee on sick leave and (financial) benefits of helping employee back to work	-	√	√	√
	Decrease stigma by advising employer how to make cancer subject of discussion	√	√	√	√
	Teach employer how to deal with uncertainty of return to work of cancer patients	-	Excluded as not feasible to realise on public website	-	-
	Information on rights and obligations according to the Dutch social security system	-	-	-	√
	List of support agencies to consult (e.g. specialised occupational physicians, reintegration agencies)	-	√	√	√ But extended
	Opportunities to share experiences with other employers	-	-	-	√
	Make direct communication between employer and treating physician possible	-	√	Excluded due to privacy and current legislation	-
	Information about grief counselling	-	-	-	Excluded because the intervention was developed for patients who are being curatively treated
**Occupational physicians** Public website	References to relevant guidelines	-	√	√	√
	Information on how to counsel employee with cancer	√	√	√	√
	General information on most common cancer types and cancer treatments	-	-	-	√
	Information on possible effects of cancer diagnosis/treatment on return to work	-	√	√	√ But extended
	Opportunity to share experiences with other occupational physicians	-	-	-	√
	List of support agencies to consult (e.g. specialised occupational physicians, reintegration agencies, etc.)	-	√	√	√ But extended
	Advice for cancer patients on how to stay in contact with workplace	-	√	√	
	Possibility for occupational physician to start support during patients' diagnostic phase	-	-	-	Excluded because it does not fit the scope of the intervention
	Possibility to access a part of the	-	-	-	Excluded

	information that patients fill in on the intervention website				because of privacy issues
	To match information to the current stage of the patient's disease	-	-	-	Excluded because of privacy issues
**General practitioners** Public website	General information on cancer diagnosis and treatment	√	√	√	√
	Information on possible effects of cancer diagnosis/treatment on return to work	√	√	√	√ But extended
	Possibility to share experiences with other general practitioners	-	-	-	√
	Information on rights and obligations according to the Dutch social security system	-	-	-	√
	List of support agencies to consult (e.g. specialised occupational physicians, reintegration agencies, etc.)	√	√	√	√ But extended
	Reference to relevant guidelines	-	√	√	√
	Information on how to counsel patients with cancer, including dos and don'ts	√	√	√	√
	Provide general practitioner with more evidence-based information on cancer and return to work	-	-	-	Excluded because it does not fit the scope of the intervention
**Specialised nurses** Access to personal and secure website	Answer questions from cancer patients	√	√	√	√
	Monitor and supervise use of the eHealth intervention	√	√	√	√
	Provide personal feedback on assignments	√	√	√	√
	Encourage cancer patients to comply with the eHealth intervention	√	√	√	√
	Information on legal and financial issues	-	-	-	√
	Quotes from other specialised nurses	√	√	√ But personalised	√

√: included. NA: not applicable. [a]: Only possible for the stakeholder the patient has invited. [b]: Fixed versus temporary employment contract.
