# Supplementary material for: Enhancing the Return to Work of Cancer Survivors: Development and Feasibility of the Nurse-Led eHealth Intervention Cancer@Work
Source: JMIR Res Protoc. 2016 Jun 10;5(2):e118. doi: 10.2196/resprot.5565 (PMC4920959; doi:10.2196/resprot.5565)
Supplement: Multimedia Appendix 2 [file resprot_v5i2e118_app2.pdf]

Multimedia Appendix 2. Design of Cancer@Work at different stages of development.

**Functionality**		**Draft 1 –** Based on literature review and interviews with experts	**Draft 2 –** Adapted on the basis of interviews with cancer survivors, occupational physicians and employers	**First version of website built** after consultation with experts on writing web text and experts on design, privacy and s	**Final website adapted** on the basis of results feasibility study
**Patients** Access to personal and secure website	Look and feel – confrontational	-	√	√	√
	Look and feel – provide perspective	-	√	√	√
	Look and feel – offer support to help them to solve specific personal problems	√	√	√	√
	Look and feel – reassure	-	√	Excluded: does not fit with tone of voice	-
	Lay-out in stages of disease (e.g. diagnosis, treatment, late effects)	-	Excluded not univocal which information about work is needed at which stage	-	-
	Combination of text, film and assignments	√	√ However, not possible due to financial constraints on the provision of film	√	√
	No prescribed use	√	√	√	√
	Look and feel – light-hearted	-	√	Excluded: does not fit with tone of voice	-
	Adapt lay-out of eHealth intervention to personality of user	-	Excluded due to financial constraints	-	-
	Possibility to enlarge the size of the text on the website for visually impaired people	-	-	-	Excluded because it does not fit the scope of the intervention
	Possibility of the text on the website being read aloud for visually impaired people	-	-	-	Excluded because it does not fit the scope of the intervention
**Employers** Public website	Look and feel – positive aspect of return to work after cancer	-	√	√	√
	Succinct information	√	√	√	√
	Headings for ‘employers’, ‘occupational physicians’ and ‘general practitioners’ are shown twice on the homepage, show only once	-	-	-	Excluded because researchers and other participants

					did not share this opinion
	Use formal form of address	-	-	-	Excluded because it does not fit the ‘tone of voice’
	Make the website look more sexy, less clinical	-	-	-	Excluded because it does not match the experts’ opinion
**Occupational physicians** Public website	Succinct information	√	√	√	√
	Change picture on the homepage because the current one looks too happy	-	-	-	Excluded because it does not meet the ‘tone of voice’ of the intervention
**General practitioners** Public website	Succinct information	√	√	√	√
**Specialised nurses** Access to personal and secure website	Succinct information	√	√	√	√

√: included.
