# Supplementary material for: Enhancing the Return to Work of Cancer Survivors: Development and Feasibility of the Nurse-Led eHealth Intervention Cancer@Work
Source: JMIR Res Protoc. 2016 Jun 10;5(2):e118. doi: 10.2196/resprot.5565 (PMC4920959; doi:10.2196/resprot.5565)
Supplement: Multimedia Appendix 3 [file resprot_v5i2e118_app3.pdf]

Multimedia Appendix 3. Features to increase compliance with Cancer@Work at different stages of development.

Features		**Draft 1 –** Based on literature review and interviews with experts	**Draft 2 –** Adapted on the basis of interviews with cancer survivors, occupational physicians and employers	**First version of website built** after consultation with experts on writing web text and experts on design, privacy and legal aspects	**Final website** adapted on the basis of results feasibility study
**Patients** Access to personal and secure website	Advice to involve a friend or family	-	√	√	√
	Send reminder	√	√	√	√
	Blended care delivered by specialised nurse which encompasses: 1) answering questions, 2) monitoring and supervising use of the eHealth intervention, 3) providing personal feedback on assignments on the eHealth intervention, and 4) encouraging patients to comply with the intervention	√	√	√	√
	Instruction video	√	√	√	√
	Advice to give employer or colleague access to personal, secure website	-	Excluded: it may be used against them	-	-
	Point out their own responsibility to do anything possible to enhance their return to work	-	Excluded: not ethical	-	-
	Structure use: show patients where they are and how much more they need to do and make appointments when they do each assignment	-	Excluded: conflicts with notion of no prescribed use	-	-
	Peer-to-peer education	-	Excluded due to financial constraints		
**Employers** Public website	Patients are able to see whether his/her employer has used the eHealth intervention	-	Excluded due to privacy constraints	-	-
	Telephone contact by an independent third party	-	Excluded due to privacy constraints	-	-
	Inclusion of official documents to meet employers' reintegration obligations	-	Excluded due to legal constraints	-	-
	Occupational physician should encourage employer to use website	-	Excluded due to privacy constraints	-	-
**Occupational physicians** Public website	Patients are able to see whether his/her occupational physician has used the website	-	Excluded due to privacy constraints	-	-

**Specialised nurses** Access to personal and secure website	Receive support from and answers to questions from an oncological occupational physician	√	√	√	√

√: included.
