# Supplementary figures and images for: Enhancing the Return to Work of Cancer Survivors: Development and Feasibility of the Nurse-Led eHealth Intervention Cancer@Work
Source: JMIR Res Protoc. 2016 Jun 10;5(2):e118. doi: 10.2196/resprot.5565 (PMC4920959; doi:10.2196/resprot.5565)

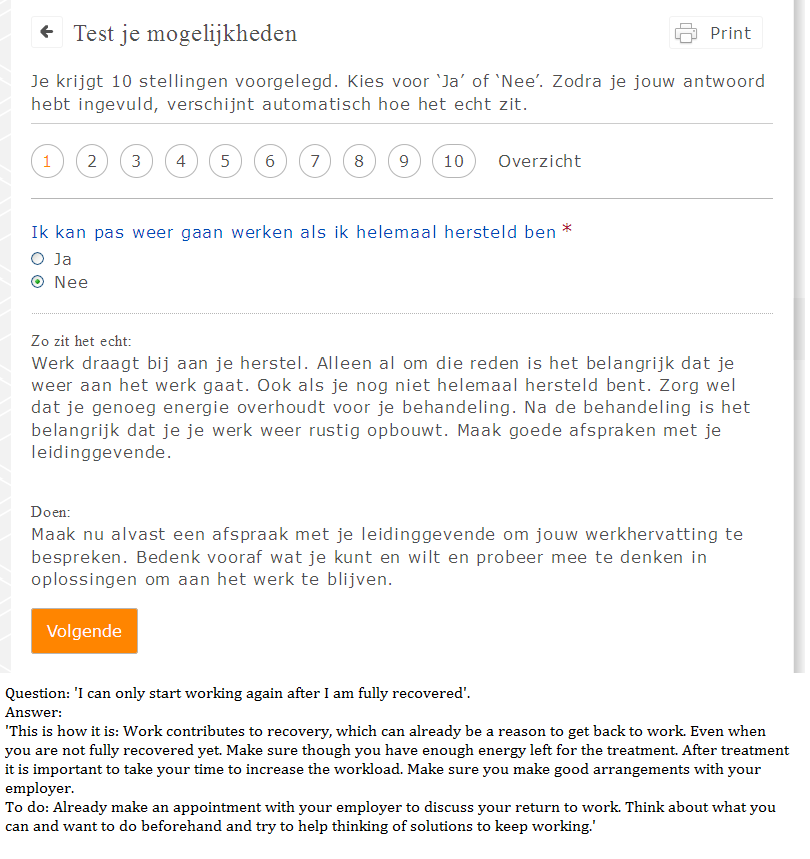

Supplement: Multimedia Appendix 4 [file resprot_v5i2e118_app4.PNG]

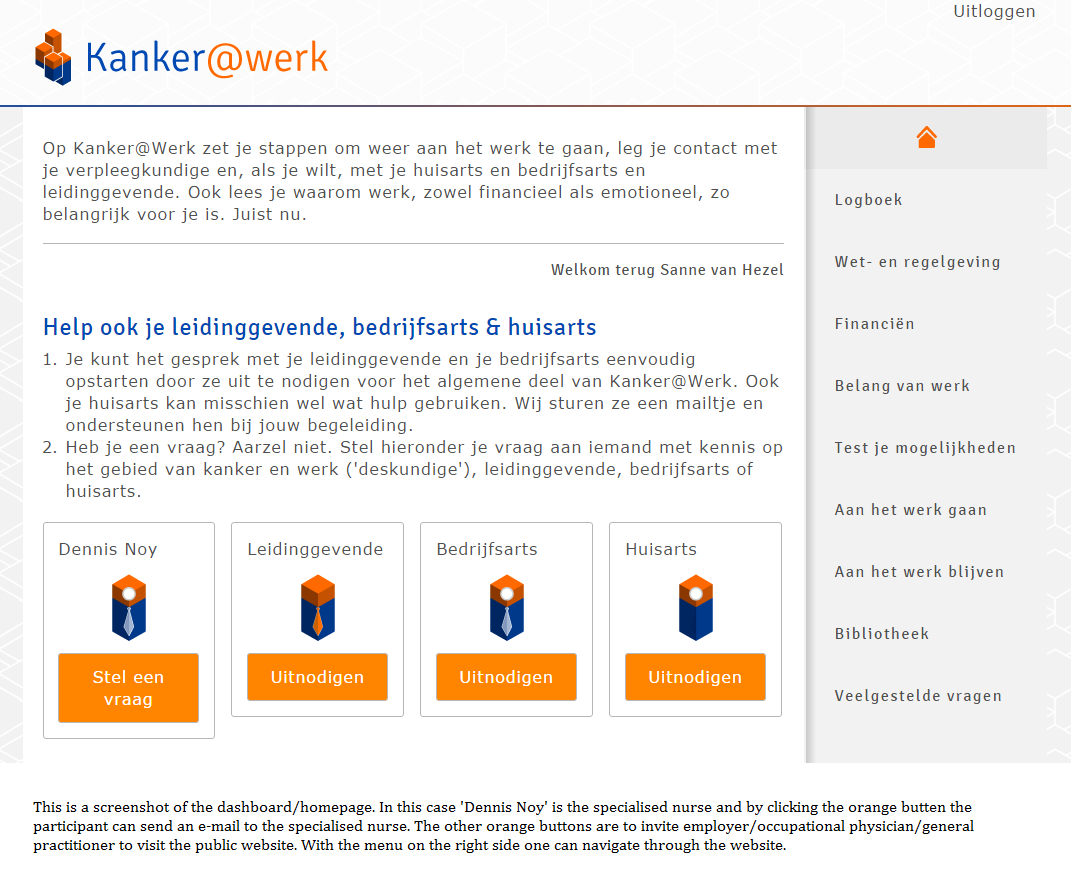

Supplement: Multimedia Appendix 5 [file resprot_v5i2e118_app5.png]
